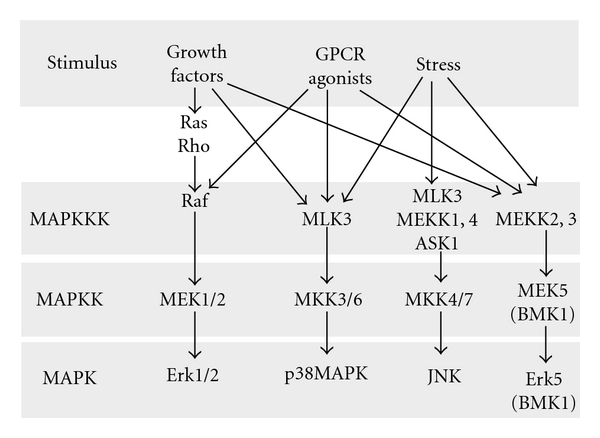# Erk in Kidney Diseases

**DOI:** 10.1155/2011/768512

**Published:** 2011-04-07

**Authors:** Denis Feliers, Balakuntalam S. Kasinath

**Affiliations:** ^1^Division of Nephrology, Department of Medicine, O'Brien Kidney Research Center, University of Texas Health Science Center, San Antonio, TX 78229, USA; ^2^South Texas Veterans Health Care System, San Antonio, TX 78229, USA

## Abstract

Acute or chronic kidney injury results from various insults and pathological conditions, and is accompanied by activation of compensatory repair mechanisms. Both insults and repair mechanisms are initiated by circulating factors, whose cellular effects are mediated by activation selective signal transduction pathways. Two main signal transduction pathways are activated during these processes, the phosphatidylinositol 3′ kinase (PI-3K)/mammalian target of rapamycin (mTOR) and the mitogen-activated protein kinase (MAPK) cascades. This review will focus on the latter, and more specifically on the role of extracellular signal-regulated kinase (ERK) cascade in kidney injury and repair.

## 1. Introduction

In acute kidney injury (AKI) and chronic kidney disease (CKD), the kidney initiates activation of signaling pathways that act as intracellular communication lines that contribute to structural and functional manifestations. Among the wide array of signaling networks activated in the kidney, those containing mammalian target of rapamycin and mitogen-activated protein kinases (MAPKs) are more commonly studied. The role of mTOR in kidney disease has been extensively reviewed recently [1, 2]. We will focus on mitogen-activated protein kinases (MAPK), and, more precisely, on Erk, one of the MAPK, in this paper. 

There are four different MAPK pathways in mammalian cells: extracellular signal-regulated kinase-1 and -2 (Erk1/2), c-Jun N-terminal kinase (JNK), p38MAPK, and extracellular signal-regulated kinase-5 (Erk5/BMK1) [3, 4]. Erk is mainly activated by mitogenic stimuli such as growth factors and hormones, and JNK and p38 are mostly activated by stress stimuli, and, are, therefore, sometimes categorized as stress kinases. Erk5 is activated by both stress stimuli and growth factors [4]. MAPKs are activated as part of three-tiered kinase cascades: they are activated by simultaneous phosphorylation on threonine and tyrosine residues by dual-specificity MAP kinase kinases (MAPKK), which are themselves activated by serine/threonine phosphorylation by MAP kinase kinase kinases (MAPKKK) [3, 4] (Figure 1). Upstream of MAPKKKs lie additional protein kinases (such as Ste20-related protein kinases) or members of the Ras and Rho families of small GTPases. An additional layer of regulation has been described in proximal tubular epithelial cells in culture, in which activation of Src by PLC*γ* lies upstream of Ras and activates Erk [5]. Pathways distinct from the kinase cascades described above can contribute to MAPK activities, and to cell specificity of MAPK activation. This paper will focus only on the role of the Erk1/2 pathway in kidney disease. 

There are greater chances of restoration of renal morphology and function after acute kidney injury (AKI) [6] than in the case of chronic kidney disease (CKD); in the latter, similar repair mechanisms may be activated although they rarely lead to complete restoration. In response to acute or chronic stress, renal cells mount a response designed to limit the extent of injury which involves activation of antiproliferative and proapoptotic genes [6]. Later, this is followed by steps aimed at repairing the injury caused by the stress and the initial response; this reparative stage involves growth factors and proliferative as well as antiapoptotic signals [6]. In AKI, these repair mechanisms often lead to restoration of renal morphology and function, but in CKD sustained activation of repair mechanisms leads to aberrant cell proliferation, cell hypertrophy, and increased extracellular matrix deposition leading to progressive renal injury.

## 2. Compensatory Renal Hypertrophy: A Physiologic Adaptation

Immediately following removal of the contralateral kidney, hyperfiltration occurs in the remaining kidney, and is followed by compensatory growth, which is due to hypertrophy of mostly tubular epithelial cells [7]. This is a physiological response to the removal of contralateral kidney. After unilateral nephrectomy, mitogenic growth factors as well as TGF*β* are upregulated in the remaining kidney. Mitogenic factors trigger the differentiated epithelial cells to exit the G0 phase and enter the cell cycle [8]. This is caused by activation of cyclin D1 and D3-activated kinases, CDK4 and CDK6 [9]. Entry into the cell cycle initiates a synthetic program that allows the cells to accumulate enough material to reach a size that permits division into two daughter cells [10, 11]. However, the concomitant increase in TGF*β* stimulates the expression of cyclin-kinase inhibitors, such p27^kip1^ and p57^kip2^ in tubular epithelial cells [12]. This, in turn, prevents activation of cyclin E-CDK2 which is necessary to pass the restriction point and enter S phase, when DNA is replicated [13]. As a consequence, tubular epithelial cells are blocked in the late G1 phase of the cell cycle when protein synthesis and accumulation of newly synthesized materials, including proteins, occur leading to cell hypertophy.

As previously described, Erk plays a crucial role in signaling by mitogenic growth factors, it is likely that Erk is important in the first phase of the hypertrophic program, when epithelial cells enter the cell cycle. Furthermore, Erk mediates upregulation of TGF*β* in tubular epithelial cells [14]. Thus, by promoting two crucial events in this process, entry into the cell cycle and upregulation of TGF*β* that prevents DNA replication, Erk plays a fundamental role in the development of compensatory kidney growth after unilateral nephrectomy.

## 3. Acute Kidney Injury

### 3.1. Ischemia/Reperfusion

Ischemia/reperfusion (I/R) injury induces both functional and morphological changes in the kidney. Necrosis, predominantly of the proximal tubule, is the hallmark of this model of renal injury. After ischemic injury, both the Erk and phosphatidylinositol 3 kinase (PI3K) signaling pathways are activated in the kidney [15, 16], notably in the region where thick ascending limbs predominate [15], whereas stress-activated kinases, p38MAPK and JNK are activated in tubular epithelial cells [15]. Erk activation is due to oxidant-induced activation of a EGF Receptor/Ras/Raf signaling cascade [16] and blockade of Erk reduces cell survival after I/R injury [15]. In addition, the renoprotective effect of preconditioning, using short period of ischemia [17] or cyclosporine A or FK506 [18] prior to an I/R insult appears to depend on decreased activation of p38MAPK and JNK, and increased activation of Erk. Similarly, inhibition of monoamine oxidase after an I/R insult potentiates Erk activation and increases proliferation but decreases necrosis of tubular cells [19]. However, a protective role for Erk was called into question by Alderliesten et al. who showed that in vivo inhibition of Erk significantly reduced renal damage after I/R injury [20].

### 3.2. Cisplatin-Induced Nephrotoxicity

Cisplatin is one of the most effective chemotherapeutic agents used for the treatment of malignant tumors, but its use is limited by its side effects, including nephrotoxicity, neurotoxicity, ototoxicity, hair loss, nausea, and vomiting [21]. Nephrotoxicity is the major dose-limiting factor during cisplatin treatment, as approximately one-third of patients experience AKI within days after cisplatin treatment [22]. Injury and death of renal tubular cells are the key pathological occurrences in cisplatin nephrotoxicity [23, 24], and Erk seems to play an important role in this process. 

In tubular epithelial cells in culture, cisplatin stimulation of Erk is mediated by an EGF-R/Src cascade [25]. Activated Erk accumulates in mitochondria following cisplatin treatment and impairs its function contributing to apoptosis; and inhibition of Erk with U0126 ameliorates mitochondrial dysfunction and apoptosis of tubular epithelial cells [26]. In mice, injection of U0126 decreases Erk activation following cisplatin administration, and offers significant renoprotection, accompanied by decreased inflammation markers, caspase 3 activity and apoptosis [27]. These data show that Erk activation mediates the renal inflammation and tubular epithelial cell apoptosis in cisplatin-induced nephrotoxicity.

## 4. Chronic Kidney Injury

### 4.1. Polycystic Kidney Disease

Autosomal dominant polycystic kidney disease (ADPKD) is one of the most common human monogenic diseases, with an incidence of 1 : 400 to 1 : 1000 [28, 29]. It is characterized by the development and gradual enlargement of multiple fluid-filled cysts within both kidneys. These cysts encroach upon and destroy normal adjacent nephrons [28]. Cyst growth and higher kidney volumes correlate with diminishing clearance function of the kidney in ADPKD [30]. Abnormalities of tubular cells lining the cysts in ADPKD include increased proliferation, increased apoptosis, abnormalities of protein sorting and polarity, and disorganization of the underlying extracellular matrix [31, 32]. In DBA2-pcy/pcy mice with polycystic kidney disease, robust Erk activation is detected in the cyst epithelium;administration of an inhibitor of the Erk pathway, PD184352, effectively reduces Erk activation and inhibits cyst-induced gain in kidney weight, cyst index and improves renal function [33]. This study underlines the important role of Erk in the formation of cysts that results from aberrant proliferation of the tubular epithelium. It also identified Erk as a potential therapeutic target in ADPKD. Since targeting mTOR with rapamycin or everolimus did not significantly ameliorate ADPKD in human subjects [34, 35], the identification of novel therapeutic targets such as Erk could be of interest.

### 4.2. Chronic Mesangioproliferative Glomerulonephritis-Induced by Anti-Thy1 Antibody

Anti-Thy1 experimental nephritis is a well-established model of experimental mesangioproliferative glomerulonephritis in the rat. Anti-Thy1 antibody binds specifically to mesangial cells and triggers complement-induced mesangiolysis, followed by rebound proliferation of mesangial cells [36]. In this model, maximum proliferation of mesangial cells is observed 6 days after injection of anti-Thy1 antibody, and it is accompanied by a significant activation of Erk and inactivation of p38MAPK in the glomerulus [37]. Treatment of rats with heparin reduces glomerular cell proliferation as well as Erk activation and restores p38MAPK activation [37]. Injection of U0126, the MEK1 inhibitor, to rats 3 days after injection of Thy1 blocks Erk activation and returns the number of proliferating glomerular cells to normal at day 6 [38]. Together, these studies demonstrate that Erk mediates and p38MAPK opposes the proliferative response in mesangioproliferative glomerulonephritis.

The role of ERK in cellular proliferation has been extensively studied. In resting conditions, Erk is anchored in the cytoplasm by its association with the microtubule network [39] and other scaffolding proteins, such as Sef [40] and PEA15 [41]. Activation of Erk by mitogens is biphasic: a first, robust, and transient phase peaks at 5–10 min and is followed by a second, weaker but more sustained phase lasting several hours [42, 43]. Nuclear translocation of Erk occurs within minutes of stimulation, is reversible upon removal of the mitogenic stimulus, and lasts throughout the G1 phase of the cell cycle [44]. Nuclear Erk is inactivated during the G1/S phase transition and is exported back to the cytosol [44]. In the nucleus, Erk phosphorylates and activates transcription factors, such as Elk1 and c-Fos, which stimulate the expression of several growth-related genes [45]. It is important to remember that Erk activation in the nucleus is required but not sufficient for successful progression through the cell cycle [8].

### 4.3. Rat Model of Progressive Membranous Nephropathy (Heymann Nephritis, PHN)

Heymann nephritis is a model of membranous nephropathy characterized by complement-dependent injury to podocytes. Injection of sublytic doses of complement (C5b-9) causes kidney damage in rats, that is restricted to podocytes. In these cells, C5b-9 causes DNA damage and cytoskeleton remodeling, along with Erk activation and upregulation of p53 and p21^cip1^ [46]. Actin cytoskeleton remodeling seems to cause localized activation of Erk and selective phosphorylation of substrates, such as cPLA2 but not Elk1 [47]. 

Inhibiting Erk in vivo in PHN worsened DNA damage in podocytes and reduced the upregulation of p21^cip1^ [46], suggesting a protective role of Erk in this model. In spite of chronic activation of Erk after overexpression of MEK, its upstream kinase, exacerbates complement-mediated podocytes in culture [47], suggesting a deleterious role for Erk. A possible explanation for this discrepancy is that overexpression of MEK causes excessive Erk activation that far exceeds what is seen in PHN in vivo and overcomes the protective role of Erk observed in vivo. These observations also emphasize the importance of context in assessing the role of Erk, while it may mediate injury response in the kidney in one context, for example, cisplatinum, it is involved in renal defense in another, for example, PHN.

### 4.4. Unilateral Ureteral Obstruction

Unilateral ureteral obstruction (UUO) in rodents generates progressive renal fibrosis due to marked renal hemodynamic and metabolic changes, followed by tubular injury and cell death by apoptosis or necrosis, with interstitial macrophage infiltration. Proliferation of interstitial fibroblasts with myofibroblast transformation leads to excess deposition of the extracellular matrix and renal fibrosis. Immediately following obstruction, a biphasic activation of Erk occurs: an early, transient phase (30 min after obstruction) of stimulation is seen in the collecting duct; this is followed by a sustained phase (4 to 7 days) in the collecting duct, the tubular epithelial cells and the cortical interstitium [48–50]. The latter phase of Erk activation has been attributed to oxidative stress [49], and its blockade prevents interstitial cell proliferation and interstitial macrophage accumulation, but not the activation of interstitial fibroblasts and renal fibrosis [50]. These results show that Erk plays a selective and limited role after UUO.

### 4.5. Diabetic Nephropathy

Characteristic morphologic changes in diabetic nephropathy (DN) include kidney hypertrophy, glomerular basement membrane thickening, and the accumulation of mesangial matrix [51, 52]. Later in the disease, progressive tubulointerstitial injury and fibrosis are observed [51, 52]. Renal enlargement, one of the first structural changes in DN, is due to the hypertrophy of existing glomerular and tubular cells rather than to cellular proliferation [51–54].

#### 4.5.1. Erk and Global Protein Synthesis

As described earlier, cellular hypertrophy is the consequence of a failure to escape the late G1 phase, when global protein synthesis takes place, and to complete the cell cycle. Cellular accumulation of protein during hypertrophy could be due both to increase in its synthesis and decrease in degradation. Stimulation of protein synthesis is due to the coordinated increase in the transcription of their respective genes, and the translation of their mRNAs; the latter is thought to be the rate-limiting step in gene expression [55, 56]. Regulation of mRNA translation can occur at the levels of both increase in efficiency of translation and capacity for translation. The former involves events occurring in the initiation and elongation phases of mRNA translation [57], whereas the latter is regulated at the level of ribosome biogenesis and assembly.

**(i) Erk in Initiation and Elongation Phases of Translation**

When a signal for increasing protein synthesis is received, the cell ramps up the process of translating the codons in mRNA into respective peptide, that is, mRNA translation. Translation occurs in three phases [56, 58]. During the initiation phase, several eukaryotic initiation factors (eIFs) assemble into two large multimeric complexes, that is, the preinitiation complex (PIC) consisting of eIF1, 1A, eIF3, eIF5, eIF2+ initiator methionyl tRNA and the 40S ribosomal subunit, and, the eIF4F complex consisting of eIF4E, eIF4G, and eIF4A [59]. The cap-binding protein eIF4E is held inactive by its binding protein, 4E-BP1, in the resting state, and is released by phosphorylation of the latter when translation is stimulated [60]. Free eIF4E undergoes phosphorylation on Ser209 and forms eIF4F complex with eIF4G and eIF4A and binds to the cap of mRNA at its 5′ end. Due to binding between eIF3 and eIF4G, a bridge is now formed between PIC and eIF4F, which brings 40S ribosomal subunit to the proximity of the mRNA. After a complex set of reactions, the 60S subunit joins 40S subunit forming the 80S ribosomal unit and the eIFs fall away from the complex but initiator methionyl tRNA remains. The 80S unit successfully localizes to the AUG codon on the mRNA, marking the end of initiation phase of translation.

All three of translation phases, initiation, elongation, and termination are exquisitely regulated [56, 57]. For instance, both initiation and elongation phases are regulated by the PI3K-Akt-mTOR signaling pathway, which ensures the coordinated activation of these two critically important events and the continuous “flow” of mRNA translation and ultimately protein synthesis. Additional layers of regulation allow fine tuning of mRNA translation. One such layer is represented by the Erk signaling pathway, which indirectly regulates the initiation phase of mRNA translation. One of Erk substrates, MAPK interacting kinase1 (Mnk1) phosphorylates eIF4E [61–63]. In contrast to mTOR-dependent phosphorylation of 4E-BP1 which is transient, Mnk1-dependent phosphorylation of eIF4E is persistent [64]. In renal epithelial cells undergoing hypertrophy under the stimulation of VEGF, Ser209 phosphorylation of eIF4E appears to be needed for increase in protein synthesis [5]. Investigation of signaling regulation showed that VEGF recruited VEGF receptor type 2 to activate phospholipase C*γ*, Src, Raf, MEK, Erk pathway in stimulating Mnk1, eIF4E phosphorylation, and protein synthesis (ibid). These data show that Erk plays an important role in increasing the efficiency of translation.

**(ii) Erk and Ribosome Biogenesis**

Cell growth, or increase in cell mass, requires a large increase in the number of ribosomes. In mammals, transcription of ribosomal DNA coding for ribosomal RNA is activated by upstream-binding factor (UBF) and selectivity factor 1. UBF activates rRNA gene transcription by recruiting RNA polymerase I to the rDNA promoter, by stabilizing binding of TIF-IB/SL1, and by displacing nonspecific DNA-binding proteins such as histone H1 [65, 66]. UBF function is regulated by phosphorylation by various kinases, such as Erk, casein kinase 2 (CK2), and cyclin-dependent kinases (CDK) [67]. Phosphorylation of Thr117 and Thr201 by Erk is essential for transcription elongation by RNA polymerase I [68, 69], whereas phosphorylation by CK2 and CDKs in the carboxy-terminal domain affect protein-protein interactions and activates rDNA transcription indirectly [70, 71]. Recent work from our lab has shown that high-glucose-induced hypertrophy and protein synthesis in glomerular epithelial cells is associated with increase in rDNA transcription (to generate ribosomal RNA) demonstrating ribosomal biogenesis. This process is dependent on UBF phosphorylation on Ser388 that was partly under the control of Erk [72]. Increase in Ser388 phosphorylation of UBF was also found in kidney parenchyma from rodent models of type 1 and type 2 diabetes, coinciding with kidney hypertrophy [72], suggesting that increased ribosomal biogenesis occurs in vivo in hypertrophic kidney during diabetes.

Ribosome assembly is an extremely complex process that involves four ribosomal RNAs (rRNAs) and approximately 80 ribosomal proteins [73]. In addition, more than 200 additional proteins and noncoding RNAs participate in the production of 60S and 40S ribosomal subunits. Ribosome assembly and activity requires posttranslational modifications of ribosomal proteins and Erk is involved in this process. In addition to generating ribosomal RNA, augmented protein synthesis involves activation of a number of proteins that are part of 40S (small, S) and 60S subunits (large, L). Ribosomal protein 6 (rpS6) and 3 (rpS3) of the 40S subunit are commonly studied.

**(iii) Ribosomal Protein S6 (rpS6)**

Ribosomal Protein S6 activation occurs during cell growth and it is a determinant of cell size [74]. Activation of rpS6 requires phosphorylation of conserved serine residues that is mediated by p70^S6K^ (S6K1) [75]. However, the fact that in mice lacking both S6K1 and S6K2, phosphorylation of rpS6 on Ser235/236 was conserved indirectly indicated that other kinases could compensate. Further studies have shown that this phosphorylation was mediated by p90^rsk^ that was itself activated by Erk [76]. Although Erk-driven rpS6 phosphorylation is functionally relevant in T-cell receptor signaling in CD8^+^ T cells [77], its significance in renal disease has not yet been established.

**(iv) Ribosomal Protein S3 (rpS3)**

Ribosomal Protein S3 possesses two independent functions. In the cytosol, it is part of the 40S subunit of the ribosome and as such participates in the initiation of mRNA translation [78]. In the nucleus, it functions as an endonuclease and is involved in DNA repair [79]. The subcellular localization of rpS3 is regulated by phosphorylation by several kinases, including Erk [80]. Phosphorylation of rpS3 on Ser42 by Erk triggers its nuclear translocation [80]. Activation of Erk can thus repress mRNA translation and stimulate DNA repair, preventing the cells from translating aberrant mRNAs. It is therefore possible that sustained activation of Erk during kidney hypertrophy in type 2 diabetes [81] could lead to a decreased availability of rpS3 for mRNA translation, thereby limiting protein synthesis and cell growth.

#### 4.5.2. Erk and Selective Protein Synthesis

Accompanying renal hypertrophy, the accumulation of extracellular matrix proteins such as type IV collagen, laminin, fibronectin, is the other cardinal manifestation in diabetic kidney disease. Progressive accumulation of matrix proteins accounts for renal fibrosis in diabetic kidney disease and is a major determinant of progressive loss of kidney function [82]. The role of the Erk pathway on the stimulation of selective synthesis of matrix proteins was investigated by our group. We reproduced the type 2 diabetic milieu (high glucose and high insulin) and studied its effect on synthesis of an important kidney extracellular matrix protein, laminin *β*1, by proximal tubular epithelial cells in culture. High glucose and high insulin, alone or in combination, triggered rapid synthesis of laminin *β*1 within 5 min of stimulation [83]. All three conditions activated the PI3K-Akt-mTOR and Erk pathways in parallel and inhibition of either pathway prevented the rapid synthesis of laminin *β*1. In insulin-treated kidney epithelial cells, Erk stimulation was downstream of PI3K, which may partly explain the common mode of regulation of laminin synthesis by both kinases [84].

## 5. Conclusion

Erk figures prominently in mediating kidney cell responses to a variety of diverse stimuli. This occurs in the physiologic setting such as compensatory kidney hypertrophy and in pathologic conditions such as models of glomerular and tubulointerstitial diseases. It should be noted that in the setting of diseases, it is not wise to generalize that Erk activation always results in tissue injury in the kidney. As reviewed above, inhibition of Erk could worsen specific kidney diseases. Thus, it is important to extend our knowledge of disease-specific regulation of Erk and then contemplate ways to modulate its activity. This requires better understanding of the role of Erk in all phases of individual kidney diseases before its modulation is planned.

## Figures and Tables

**Figure 1:**